# Adsorption Affinities of Small Volatile Organic Molecules on Graphene Surfaces for Novel Nanofiller Design: A DFT Study

**DOI:** 10.3390/molecules28227633

**Published:** 2023-11-16

**Authors:** Francesco Moriggi, Vincenzina Barbera, Maurizio Galimberti, Giuseppina Raffaini

**Affiliations:** Department of Chemistry, Materials, and Chemical Engineering “Giulio Natta”, Politecnico di Milano, Via Luigi Mancinelli 7, 20131 Milano, Italy; francesco.moriggi@polimi.it (F.M.); vincenzina.barbera@polimi.it (V.B.)

**Keywords:** adsorption, surface modification, DFT, nano-sized carbon allotropes, graphene, carbon nanotubes, supramolecular interactions, pyrrole methodology

## Abstract

The adsorption of organic molecules on graphene surfaces is a crucial process in many different research areas. Nano-sized carbon allotropes, such as graphene and carbon nanotubes, have shown promise as fillers due to their exceptional properties, including their large surface area, thermal and electrical conductivity, and potential for weight reduction. Surface modification methods, such as the “pyrrole methodology”, have been explored to tailor the properties of carbon allotropes. In this theoretical work, an ab initio study based on Density Functional Theory is performed to investigate the adsorption process of small volatile organic molecules (such as pyrrole derivatives) on graphene surface. The effects of substituents, and different molecular species are examined to determine the influence of the aromatic ring or the substituent of pyrrole’s aromatic ring on the adsorption energy. The number of atoms and presence of π electrons significantly influence the corresponding adsorption energy. Interestingly, pyrroles and cyclopentadienes are 10 kJ mol^−1^ more stable than the corresponding unsaturated ones. Pyrrole oxidized derivatives display more favorable supramolecular interactions with graphene surface. Intermolecular interactions affect the first step of the adsorption process and are important to better understand possible surface modifications for carbon allotropes and to design novel nanofillers in polymer composites.

## 1. Introduction

The adsorption process on solid surfaces plays a critical role in numerous scientific disciplines, ranging from chemistry and physics to materials science and nanotechnology [1,2,3,4,5,6,7,8]. Many phenomena can occur when molecules adsorb on solid surface. The ability to understand and manipulate the interactions between molecules and surfaces opens up avenues for designing novel materials with tailored surface properties and optimizing the performance of existing systems [9,10,11,12,13,14,15]. While experimental techniques provide valuable information about surface adsorption, a deeper understanding at the atomic level requires the use of numerical simulations that can help us study the possible mechanisms and dynamics driving these processes [16,17,18,19,20,21,22].

Density Functional Theory (DFT) calculations together with molecular mechanics (MM) and molecular dynamics (MD) methods can provide information that strongly complements experimental studies [23,24,25,26,27,28,29,30,31,32,33,34,35,36,37,38]. MM and MD simulations are interesting tools to describe both the bulk and surface properties of materials at the atomistic level [39,40,41,42,43,44,45,46,47]. The adsorption process of proteins, peptides, and small molecules can be investigated on the external surfaces of graphite and carbon allotropes in general. The comparisons between theoretical results and experimental data are interesting, such as those concerning favorable van der Waals interactions between graphene allotropes and proteins, an important aspect for biomaterials in contact with the blood [43]. The same favorable interactions explain the possible solubilization of carbon nanotubes synthesized in an amorphous phase due to protein adsorption on the external surface. Their solubilization is an important technology aspect to prepare aligned fibers in polymeric matrices of composite materials [47,48,49].

Considering the adsorption of a single molecule on a solid surface, Density Functional Theory has emerged as an interesting tool for studying the ground-state properties of condensed matter systems, particularly with regard to how surface interactions influence the electronic distribution in the molecular orbitals of the volatile organic molecules near a solid surface [50,51,52,53]. DFT offers a rigorous framework based on quantum mechanics, enabling accurate predictions of adsorption sites, binding energies, and electronic properties that closely align with experimental observations, allowing researchers to gain insights into the structural, electronic, and energetic aspects of adsorption, thus helping to clarify the fundamental principles that govern these phenomena [23,50,51,52,53,54].

In recent years, there has been a growing interest in surface modification techniques aiming to improve the properties of fillers used in advanced technological applications [55]. One prominent area where surface modification has shown significant potential is in the development of polymer composites for the tire industry [56,57,58]. By modifying the surfaces of fillers, scientists can achieve substantial improvements in mechanical strength, thermal stability, electrical conductivity, and other desired characteristics, thereby pushing the boundaries of material performance [59,60,61,62]. In this context, nano-sized carbon allotropes, such as graphene and carbon nanotubes, have emerged as promising candidates due to their exceptional properties [63,64]. Carbon allotropes display a remarkable combination of attributes that make them highly attractive as fillers in polymer composites [65,66,67,68,69,70,71]. Their large surface area provides an extended contact interface with the surrounding polymer matrix, enabling efficient load transfer and reinforcing the mechanical properties of the composite [66,67,68]. Additionally, carbon allotropes exhibit exceptional thermal and electrical conductivity, facilitating heat dissipation and electrical conduction pathways within the material. Furthermore, their incorporation into composites allows for a reduction in the volume ratio of fillers compared to traditional alternatives, which can lead to lighter and more cost-effective materials [53]. Given these advantages, tailoring the properties of carbon allotropes has become a topic of considerable scientific interest, driving the exploration of various surface modification methods [69,70,71,72,73,74].

Among the diverse approaches investigated, one particularly efficient and reliable procedure is known as the “pyrrole methodology”, which involves the covalent modification of carbon allotrope surfaces using *N*-substituted pyrrole molecules [75,76], a method that enables the introduction of desirable functionalities onto the carbon allotrope surface. The grafting process begins with the initial adsorption of *N*-substituted pyrrole molecules onto the sp^2^ carbon surface, which then undergoes oxidation and subsequent Diels–Alder cycloaddition, forming covalent bonds with the edges of the carbon allotrope plane. The adsorption and grafting mechanisms of the pyrrole methodology are governed by a complex interplay of supramolecular interactions, which dictate the stability, structure, and properties of the modified surface [77]. This approach has been employed to produce novel fillers in order to improve the mechanical properties of polymer nanocomposites [78,79].

Motivated by the potential of the pyrrole methodology and the need for a detailed understanding of the adsorption process, our study employed ab initio simulations based on DFT to gain deeper insights into the initial steps of the pyrrole methodology and investigate the adsorption behaviors of various compounds on carbon allotropes. Our objectives encompassed not only determining the optimal computational parameters for the simulations but also exploring the interactions between pyrrole molecules and graphene surfaces. Furthermore, we sought to examine the effects of substituents and oxidation on the adsorption process, allowing us to understand how different modifications influence the stability and reactivity of this system.

To broaden the scope of our investigation, we expanded our calculations to include other compounds, including alkanes, cyclopentanes, pyrrolidines, and cyclopentadiene derivatives. By exploring a broad range of molecular species, we aimed to uncover the underlying factors governing adsorption and shed light on the role of dispersion and π-π interactions in stabilizing these systems on graphene surfaces, an approach that has already been verified in the recent literature [80,81,82,83]. The structures of the systems were studied by calculating their adsorption energies and generating charge difference density plots. These detailed analyses allowed us to obtain a comprehensive understanding of the supramolecular interactions at play and their influence on the adsorption phenomena occurring on carbon allotrope surfaces. Table 1 shows a list of the compounds adsorbed on the pristine graphene samples studied. The pyrrole compounds used to develop the “pyrrole methodology” were obtained from the Paal Knorr reaction [84,85] of a primary amine with 2,5-hexanedione (HD). This synthetic pathway gave the chance to start from a biobased chemical. Indeed, HD was prepared through the ring opening reaction of 2,5-dimethylfuran, also by using a two-step one pot process [86,87]. The use of HD led to pyrrole molecules with two methyl groups in the alpha positions of the ring. In Section 2.4, theoretical results about 1,2,5-trimethylpyrrole and its oxidized derivatives on a pristine graphene surface will be discussed. The use as reinforcing fillers of sp^2^ carbon allotropes, mainly carbon black, functionalized by means of the “pyrrole methodology” with pyrrole compounds as the ones studied in the research here reported, improved the properties of elastomeric composites for a large-scale application such as the one in tyre compounds [87,88]. The development on an industrial scale was announced by a major player in the tyre field [89]. This study is aimed at giving a contribution to the development of the “pyrrole methodology”, elucidating its first step.

## 2. Results and Discussion

In this section, the results about the adsorption of the different aliphatic and aromatic compounds reported in Table 1 on the pristine graphite surface are reported and discussed.

### 2.1. Adsorption of Linear Alkanes on the Pristine Graphene Surface

**Figure 1:**
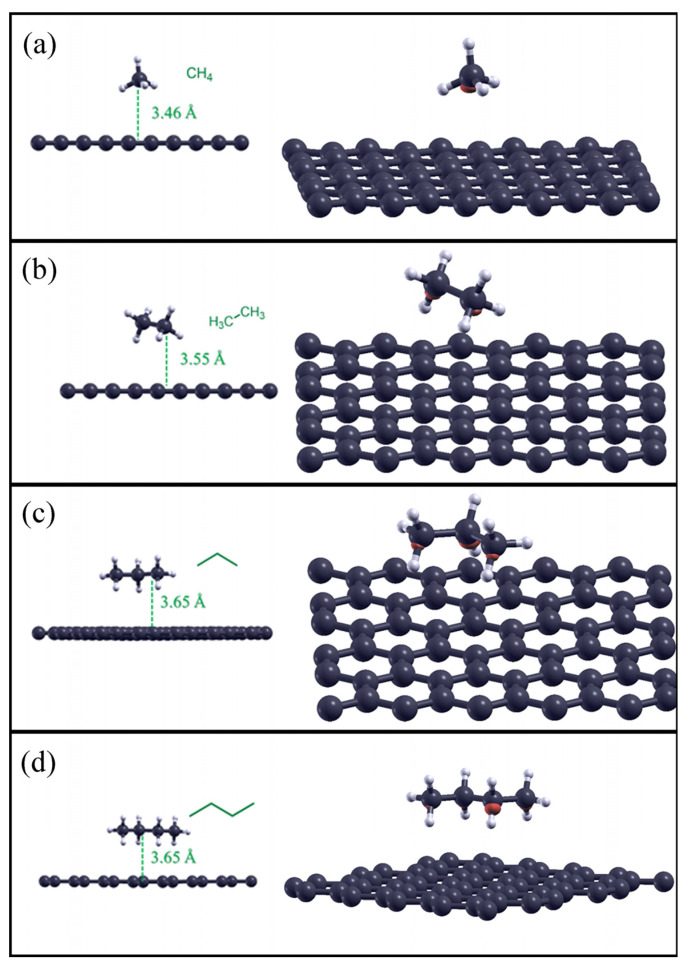
Optimized structures for the studied alkanes adsorbed on the pristine graphene surface (on the left), and their charge density difference plots (iso-surface value: 0.0003) on the surface (on the right). Methane adsorbed on graphene is reported in panel (**a**), ethane in panel (**b**), propane in panel (**c**), butane in panel (**d**), respectively.

As described in the Materials and Methods section, linear alkane compounds such as methane, ethane, propane, and butane were considered close to the pristine surface of graphene. The optimized structures are shown to the left of all four panels in Figure 1, while the adsorption distances and adsorption energies are listed in Table 2. As seen in the latter, the adsorption distances ranged from 3.46 Å (methane) to 3.65 Å (ethane), and the adsorption energies increased with increasing carbon atoms in the alkyl chain, indicating increased supramolecular interactions due to the forces of dispersion between the molecule and the graphene surface.

The charge density difference iso-surfaces (value = 0.0003) shown to the right of all four panels in Figure 1 indicate the regions where there is charge transfer, and this information is useful for representing the nature of bonding between the elements present in the simulation. The small red clouds present for all adsorbents indicate a slight increase in charge density (charge per unit volume) pointing toward the graphene surface in the direction of the C–H bond. The red clouds are more evident for propane and butane in Figure 1c,d, respectively.

Regarding the ground-state geometries studied, upon considering the plane defined by all carbon atoms in the propane and butane chains, it can be gleaned that this plane is parallel to the graphene plane. As regards the interaction energy, we can observe that as the number of carbon atoms in the chain increases, the interactions with the solid surface increase proportionally, indicating a stabilizing effect due, as mentioned previously, to the favorable dispersion interactions between the C–H bonds and the graphene surface.

### 2.2. Adsorption of Saturated Cyclic Compounds on the Pristine Graphene Surface

The results relating to the saturated cyclic systems reported in Table 1 are presented and discussed in this section, considering both cyclopentane and pyrrolidine compounds.

#### 2.2.1. Adsorption of Cyclopentane Compounds on the Pristine Graphene Surface

**Figure 2:**
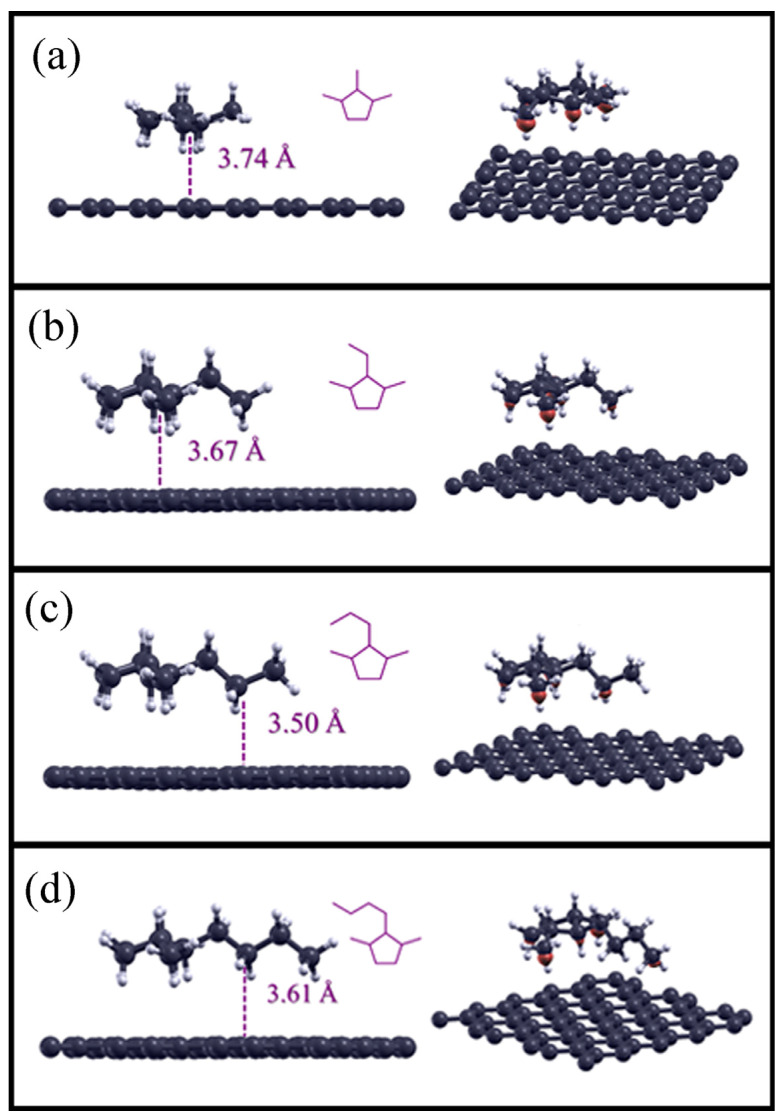
Optimized structures for the cyclopentane compounds adsorbed on the pristine graphene surface (on the left), and the charge density difference plots (iso-surface value: 0.0003) of the alkane compounds on the surface (on the right). 1,2,3-trimethylcyclopentane adsorbed on graphene is reported in panel (**a**), 2-ethyl-1,3-dimethylcyclopentane in panel (**b**), 2-propyl-1,3-dimethylcyclopentane in panel (**c**), 2-butyl-1,3-dimethylcyclopentane in panel (**d**), respectively.

As described in the Materials and Methods section, cyclopentane compounds were considered adsorbed on the pristine graphene surface. The ground-state structures are shown on the left in Figure 2, while the adsorption distances and the adsorption energies are reported in Table 3. 

Compared to the previous results, considering the structures in the ground-state ranging from 1,2,3-trimethylcyclopentane to 2-butyl-1,3-dimethylcyclopentane, we observed a different arrangement of the structure of the alkyl residue -R with respect to the graphene plane: in fact, if we also consider for these structures the plane defined only by the carbon atoms linked to carbon 2 of the cyclopentane, this plane is now arranged perpendicular to the graphene surface, as reported in the panels on the left of Figure 2. The distances of carbon atoms closer to the graphene surface varied from 3.74 Å in 1,2,3-trimethylcyclopentane to 3.50 Å in 2-propyl-1,3-dimethylcyclopentane.

Concerning the strength of the interactions, the adsorption energies were higher when increasing the carbon atoms in linear chain, indicating, as with the alkane compounds, increased supramolecular interactions due to dispersion forces between these cyclopentane derivatives and the graphene surface.

Regarding the charge difference density plots (iso-surface value: 0.0003) shown on the right in all panels in Figure 2, and regarding the linear alkanes, the red clouds present for all the adsorbates indicate an increase in charge density that points toward the graphene surface. 

As mentioned previously, regarding the geometry of the interactions in the calculated ground state, the alkyl chains that start from the cyclopentane are perpendicular to the graphene surface, and the adsorbate atoms that are close to the latter seem to contribute more to the overall supramolecular interactions. Moreover, because cyclopentanes are *sp^3^*-hybridized, there are no planar geometry constraints; thus, the molecules, after adsorption, adjust themselves to maximize the interaction forces due to C–H bonds and the graphene surface.

#### 2.2.2. Adsorption of Pyrrolidine Compounds on the Pristine Graphene Surface

**Figure 3:**
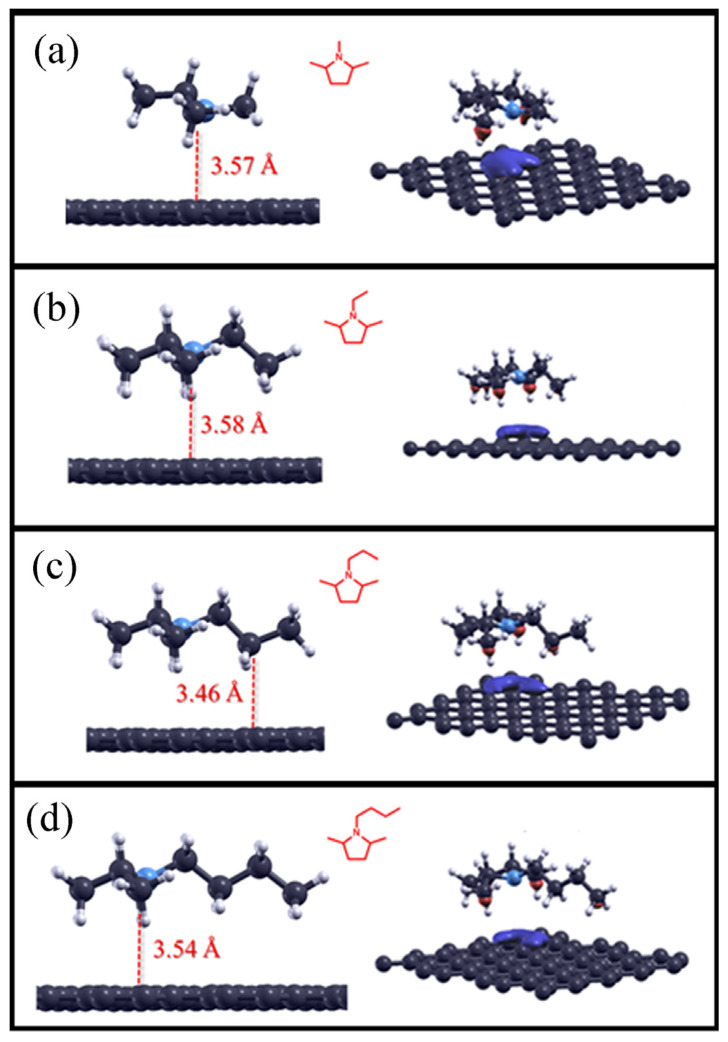
Optimized structures for pyrrolidine compounds adsorbed on the pristine graphene surface (on the left), and the charge density difference plots (iso-surface value: 0.0003) of alkane compounds on the surface (on the right). 1,2,5-trimethylpyrrolidine adsorbed on graphene is reported in panel (**a**), 1-ethyl-2,5-dimethylpyrrolidine in panel (**b**), 1-propyl-2,5-dimethylpyrrolidine in panel (**c**), 1-butyl-2,5-dimethylpyrrolidine in panel (**d**), respectively.

As described in the Materials and Methods section, pyrrolidine derivatives adsorbed on the pristine graphene surface were studied. The lowest total energy structures are reported in the panels on the left in Figure 3, while the adsorption distances and their adsorption energies are listed in Table 4. Compared to the previous results, the calculated distances of pyrrolidine compounds in the calculated ground state were, on average, smaller, ranging from 3.46 Å to 3.58 Å for 1-propyl-2,5-dimethylpyrrolidine and 1-ethyl-2,5-dimethylpyrrolidine, respectively. Using the same methodologies for the analysis of the theoretical results, the adsorption energies were calculated, and their values were found to be lower, and therefore more stable in the system, for molecules with a greater number of carbon atoms, which were comparable in size to compounds of cyclopentane. 

Considering the graphs of the charge density differences for the pyrrolidines (see the right side in all panels in Figure 3), one can see, as in the previous case, the positive (red) contribution to the charge transfer between the carbon atoms and the surface of graphene. Compared to saturated homoatomic systems (e.g., alkanes and cyclopentanes), however, the nitrogen atom appeared to deplete the graphene surface, as can be gleaned from the blue cloud under the pyrrolidine ring. This could be explained by the fact that nitrogen possesses a lone pair of electrons, which, in addition to the dispersion forces between the C–H bonds and the graphene surface, can interact with the graphene surface via a π-type bond.

### 2.3. Adsorption of Unsaturated Cyclic Compounds on the Pristine Graphene Surface

The results regarding the unsaturated cyclic systems reported in Table 1 are presented and discussed in this section, considering both cyclopentadiene and pyrrole compounds.

#### 2.3.1. Adsorption of Cyclopentadiene Compounds on Pristine Graphene Surface

**Figure 4:**
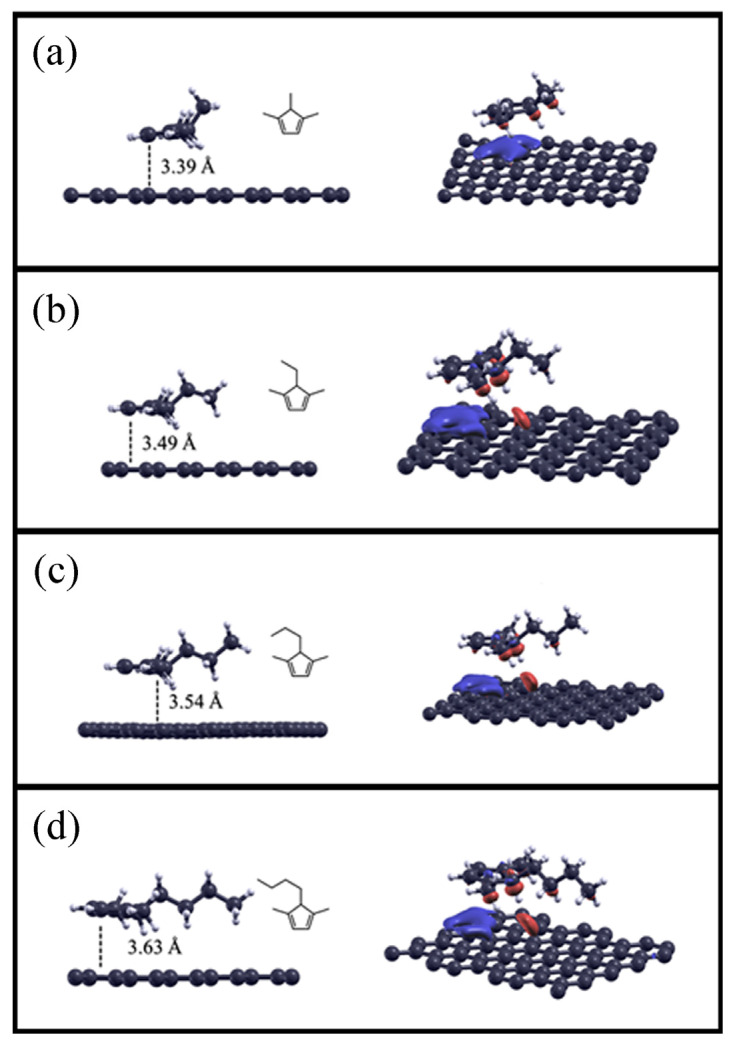
Optimized structures for cyclopentadiene compounds adsorbed on the pristine graphene surface (on the left), and the charge density difference plots (iso-surface value: 0.0003) of alkane compounds on the surface (on the right). 1,4,5-trimethylcyclopenta-1,3-diene adsorbed on graphene is reported in panel (**a**), 5-ethyl-1,4-dimethylcyclopenta-1,3-diene in panel (**b**), 5-propyl-1,4-dimethylcyclopenta-1,3-diene in panel (**c**), 5-butyl-1,4-dimethylcyclopenta-1,3-diene in panel (**d**), respectively.

As described in the Materials and Methods section, cyclopentadiene derivatives were adsorbed on the pristine graphene surface. The optimized structures are reported on the left in all panels in Figure 4, while the adsorption distances and calculated adsorption energies are shown in Table 5.

We can observe that the studied cyclopentadiene compounds, due to *sp^2^* hybridization, have a planar ring that is positioned parallel to the graphene surface to maximize the interaction area, with the distance d increasing as the number of atoms adsorbed increases.

Compared to the results for the previously reported saturated compounds, the calculated adsorption energies are higher but still follow the same trend, ranging from −51.5 kJ mol^−1^ to −67.7 kJ mol^−1^ for 1,4,5-trimethylcyclopenta-1,3-diene and 5-butyl-1,4-dimethylcyclopenta-1,3-diene, respectively. 

In the charge density difference plots shown in all panels on the right in Figure 4, we can observe that there are two contributions to the adsorption of cyclopentadiene derivatives on the graphene surface. Initially, dispersion interactions are significant, such as in alkane compounds, as indicated by the increase in charge density (red cloud) in the direction of the C–H bonds toward the graphene surface. Furthermore, the contribution of π interactions between the molecular π orbitals of the cyclopentadiene rings and the delocalized π orbitals of the graphene surface is important, highlighted in this case by the charge depletion (blue cloud) on the graphene surface.

#### 2.3.2. Adsorption of Pyrrole Compounds on the Pristine Graphene Surface

**Figure 5:**
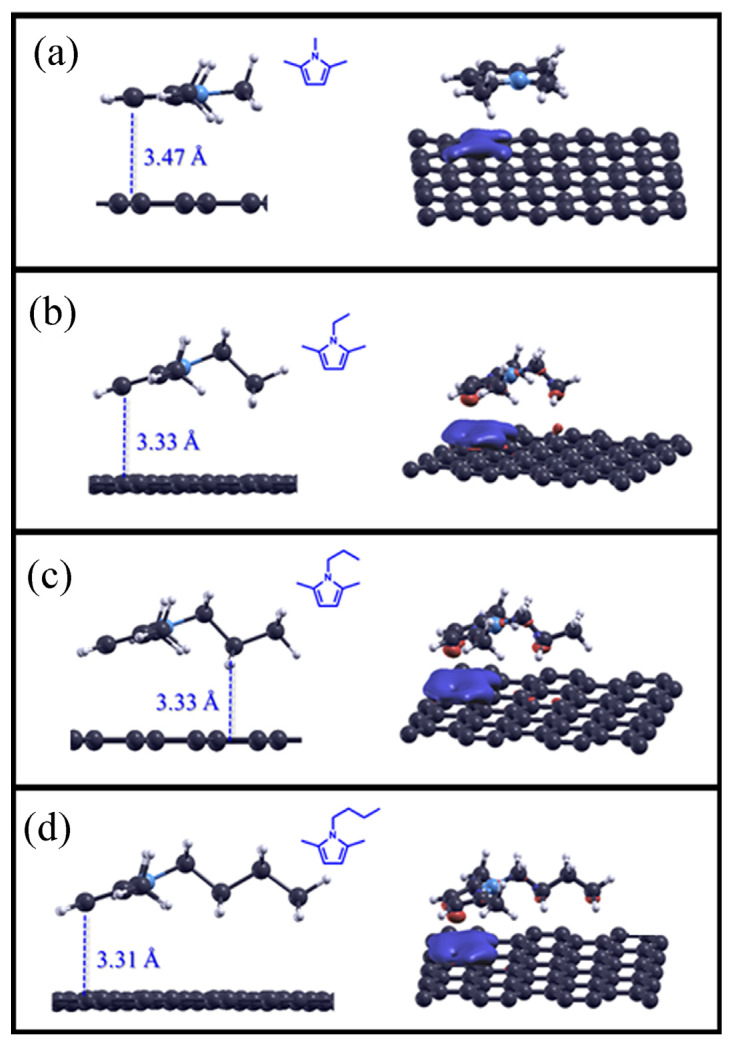
Optimized structures for pyrrole derivatives adsorbed on the pristine graphene surface (on the left), and the charge density difference plots (iso-surface value: 0.0003) of alkane compounds on the surface (on the right). 1,2,5-trimethylpyrrole adsorbed on graphene is reported in panel (**a**), 1-ethyl-2,5-dimethylpyrrole in panel (**b**), 1-propyl-2,5-dimethylpyrrole in panel (**c**), 1-butyl-2,5-dimethylpyrrole in panel (**d**), respectively.

As described in the Materials and Methods section, the theoretical results regarding the pyrrole derivatives adsorbed on the pristine graphene surface are shown and discussed in this section. The optimized structures can be seen in Figure 5, while the adsorption distances and calculated adsorption energies are listed in Table 6.

Unlike unsaturated cyclopentadiene compounds, pyrroles are aromatic compounds, so the delocalized molecular orbitals in the ring constraint fix the carbon attached to the nitrogen in the same plane. This causes molecules that have a longer chain than that of 1,2,5-trimethylpyrrole to be slightly inclined and not perfectly parallel to the graphene surface, thus resulting in shorter adsorption distances between the molecule and the latter.

Even for these systems, the adsorption energy (E*_ads_*) increases as the number of atoms in the molecule increases, specifically in the substituent of the linear alkyl chain, with values ranging from −55.4 kJ mol^−1^ to −66.3 kJ mol^−1^ for 1,2,5-trimethylpyrrole and 1-butyl-2,5-dimethylpyrrole, respectively.

In the graphs of the charge density differences reported on the right-hand panels in Figure 5 (iso-surface values: 0.0003), specifically regarding the cyclopentadiene derivatives, we can observe that two different contributions to the adsorption strength are important: the first contribution increases the density of charge between carbon atoms facing the direction of the C-H bond due to dispersion interactions, and the second contribution induces a charge depletion on the graphene surface due to π–π interactions.

**Figure 6:**
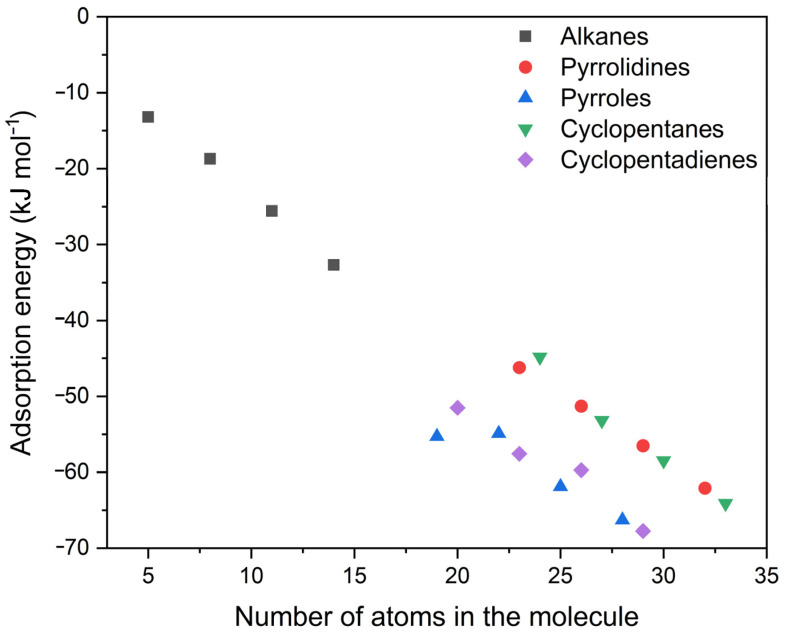
Adsorption energies of compounds adsorbed on the pristine graphene surface as a function of the number of atoms in the specific organic molecule.

In Figure 6, the adsorption energy on the pristine graphene surface has been plotted as a function of the number of atoms in the studied molecule. As expected, a linear correlation was found when increasing the number of carbon atoms present (see Table 7). Since there are no covalent or ionic bonds present between the molecule and the carbon substrate, the only forces exerted between these two systems are, as previously mentioned, due partly to dispersion and partly, if present in the case of adsorbates with free electron bonds in p-type orbitals, to π–π interactions. Further theoretical studies based on molecular mechanics and dynamics method on the role of the van der Waals contributions using a simulation previously proposed protocol [90] are an ongoing work.

For alkane and cyclopentane compounds, which lack these mechanisms, a possible adsorption mode is through the dispersion bond, while pyrrolidines, cyclopentadienes, and pyrroles, which have π electrons, can interact with the surface through π–π interactions. At the same number of atoms, cyclopentadiene and pyrrole compounds showed better stability and a gain of about 10 kJ mol^−1^ compared to their saturated counterparts, cyclopentanes and pyrrolidines, respectively. Interestingly, the pyrrole compounds, which interact better with the graphene surface, show a negative and greater slope in the best linear fit as reported in Table 7.

### 2.4. Adsorption of 1,2,5-Trimethylpyrrole and Its Oxidized Derivatives on Pristine Graphene Surface

**Figure 7:**
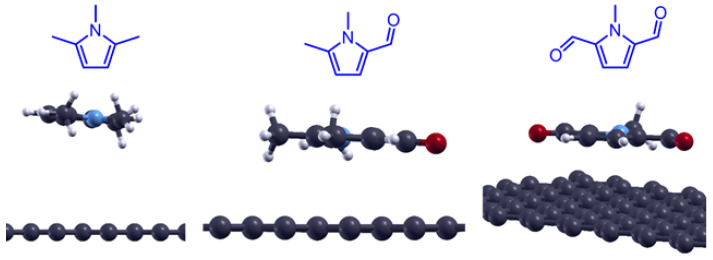
Optimized structures for TMP, TMP-CHO, and TMP-2CHO adsorbed on the pristine graphene surface.

**Figure 8:**
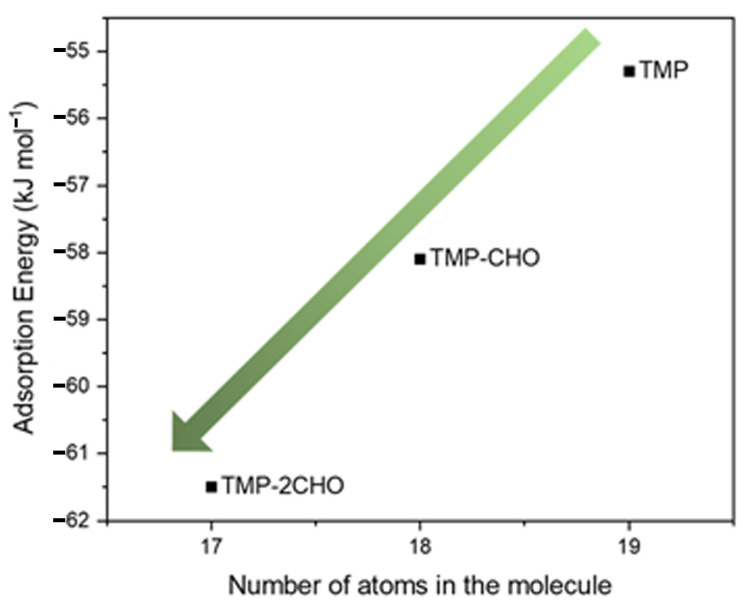
Adsorption energies for TMP, TMP-CHO, and TMP-2CHO on the pristine graphene surface versus the number of atoms in the molecule.

To study the effect of oxidation, which, as described in the introduction, is a key step for the cycloaddition of pyrrole molecules onto carbon allotropes [77,91], 1,2,5-trimethylpyrrole and its oxidized derivatives were adsorbed on the pristine graphene surfaces and their adsorption energies calculated. In Figure 7, ground-state energy structures for TMP, TMP-CHO, and TMP-2CHO can be seen. For all the compounds, the value of the adsorption distance was found to be 3.46 Å. Figure 8 shows the adsorption energies as a function of the number of carbon atoms in the organic adsorbates. As can be seen from the E*_ads_* values, the presence of an aldehyde group favors the adsorption process, with a decrease of about 3 kJ mol^−1^ per aldehyde. This means that the oxidation of pyrrole compounds enhances the supramolecular interactions between the latter and the graphene surface, as the oxygen increases the number of π interactions with the conjugated graphene system. Regarding the effect of oxidation, we can conclude that the presence of aldehyde groups improved the adsorption energy for 1,2,5-trimethylpyrrole on the pristine graphene surface.

## 3. Materials and Methods

The specific initial geometries of volatile organic compounds near graphene layer were obtained after molecular mechanics and molecular dynamics simulations using a simulation protocol adopted in previous work [90] about the adsorption process on graphite, graphene surface. These data will be published in a future paper related to the adsorption of single volatile compounds on graphene surface and at larger concentration.

Optimal adsorption configurations were derived using pw.x software, part of the Quantum ESPRESSO suite of codes [92]. This package is widely used to simulate the behavior of materials and molecules, and pw.x software [93] specifically performs Density Functional theory (DFT) calculations related to the electronic structures of materials. A non-empirical generalized gradient approximation functional, namely, Perdew–Burke–Ernzerhof (PBE), was used [94], and standard solid-state ultrasoft pseudopotentials were employed to process the electron–ion interactions for all the atoms [95]. The kinetic energy cutoff of the plane wave basis set was fixed at 60 Ryd. For pristine graphene systems, a 3 × 3 × 1 Monkhorst–Pack set was used to sample the Brillouin zones, while the other simulations were performed at the Γ point. To account for dispersion (Van der Waals) interactions, which, as mentioned in the introduction, are important in describing physisorption on graphene-based systems, the “DFT-D of Grimme” algorithm was used [96].

To model the pristine graphene sheet, a periodic unit cell with dimensions of 6 × 5 × 1 was built. The unit cell contained 60 carbon atoms, and the lattice parameters were set to 12.30 Å, 12.78 Å, and 20.33 Å in the a, b, and c directions, respectively. To prevent any interaction that could introduce errors in the analysis, the c parameter (the height of the box in which the calculation was carried out) was adjusted to 20 Å, ensuring a sufficient separation between the graphene layers.

Regarding the different types of small organic molecules adsorbed on the surface of graphene, the analyzed compounds and their chemical structures are listed in Table 1. For the R group, linear alkyl chains were investigated by increasing the number of carbon atoms from methane to butane; the other molecules were cyclic saturated with cyclopentane, pyrrolidine, unsaturated cyclic cyclopentadiene, and pyrrole.

With regard to adsorption energy, it is important to highlight that to quantify the interaction force between the adsorbate and the investigated surface, adsorption energy (*E_ads_*) was calculated as follows:

*E_ads_* = *E*_(*S*+*A*)_ − *E_A_* − *E_S_*

Above, *E*_(*S*+*A*)_ is the total energy of the adsorbate/surface system, *E_A_* is the total energy of the molecule calculated with the same cell and electronic parameters of the whole system, and *E_S_* is the total energy of the investigated surface. As a quantity strictly connected to the thermodynamics of adsorption, the more negative the value, the more favorable the process.

Charge density difference plots were used to visualize the charge transfer and charge disequilibria between atoms from the adsorbate, and surface charge density difference (CDD) maps were calculated using the pp.x software contained in the Quantum ESPRESSO Suite. Δρ was calculated using the following formula:

Δρ = ρ_(S+A)_ − ρ_A_ − ρ_S_

Above, ρ_(S+A)_ is the charge density of the adsorbate/surface system, ρ_A_ is the charge density of the molecule (calculated with the same cell and electronic parameters of the whole system), and ρ_S_ is the charge density of the investigated surface. Following this methodology, Δρ (or, more specifically, its iso-surface) shows the variations in the charge density of the graphene–adsorbate system. In the figures, a difference in charge density is shown in red on an iso-surface on which the accumulation of charge is represented, while blue indicates depletion [97,98].

## 4. Conclusions

The DFT study of pyrroles and pyrrole derivatives adsorbed on graphene surface revealed that adsorption process is predominantly governed by dispersion forces and π–π bonding interactions. The number of atoms in the molecules considered and the presence of π electrons significantly influence the corresponding adsorption energy. A linear dependence of the adsorption energy is found as a function of the number of atoms in the adsorbed molecules. The pyrrole compounds display more favorable supramolecular interactions with graphene surface. In particular, at the same number of atoms in contact with the graphene surface pyrroles and cyclopentadienes are 10 kJ mol^−1^ more stable than the corresponding unsaturated ones. Furthermore, the presence of aldehydic groups in pyrrole derivatives improves the adsorption energy which is therefore more negative. This study provides valuable insights into supramolecular interactions and their influence on the first step of the adsorption process, contributing to the fundamental understanding of surface modifications for carbon allotropes and their applications in polymer composites.

Overall, the findings of this study offer valuable insights into the complex mechanisms governing adsorption on carbon allotrope surfaces. By elucidating the significant roles of dispersion forces and π–π bonding interactions, this research study contributes to a comprehensive understanding of surface modifications for carbon allotropes and their potential applications in the field of polymer composites. Further study on the adsorption process of these molecules on the graphene surface as single molecules and at higher concentrations will be performed using molecular mechanics and molecular dynamics methods to better understand the contribution of van der Waals interactions in the adsorption process in a more complex supramolecular structure as in previous work [90].

## Figures and Tables

**Table 1:** A list of the studied compounds adsorbed on pristine graphene.

Compound	Structure Type	Structure
Alkane	Linear	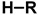
Cyclopentane	Saturated cyclic	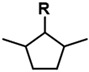
Pyrrolidine		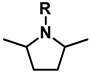
Cyclopentadiene	Unsaturated cyclic	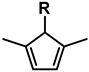
Pyrrole		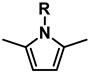

**Table 2:** Adsorption distances and energies for alkane compounds adsorbed on the pristine graphene surface.

Compound	Distance, *d* (Å)	E*_ads_* (kJ mol^−1^)
Methane	3.46	−13.2
Ethane	3.55	−18.7
Propane	3.65	−25.6
Butane	3.65	−32.7

**Table 3:** Adsorption distances and energies for cyclopentane compounds adsorbed on the pristine graphene surface.

Compound	Distance, *d* (Å)	E*_ads_* (kJ mol^−1^)
1,2,3-trimethylcyclopentane	3.74	−48.1
2-ethyl-1,3-dimethylcyclopentane	3.67	−53.2
2-propyl-1,3-dimethylcyclopentane	3.50	−58.5
2-butyl-1,3-dimethylcyclopentane	3.61	−64.1

**Table 4:** Adsorption distances and energies for pyrrolidine compounds adsorbed on the pristine graphene surface.

Compound	Distance, *d* (Å)	E*_ads_* (kJ mol^−1^)
1,2,5-trimethylpyrrolidine	3.57	−46.2
1-ethyl-2,5-dimethylpyrrolidine	3.58	−51.3
1-propyl-2,5-dimethylpyrrolidine	3.46	−56.5
1-butyl-2,5-dimethylpyrrolidine	3.54	−62.1

**Table 5:** Adsorption distances and energies for cyclopentadiene compounds adsorbed on the pristine graphene surface.

Compound	Distance, *d* (Å)	E*_ads_* (kJ mol^−1^)
1,4,5-trimethylcyclopenta-1,3-diene	3.57	−46.2
5-ethyl -1,4-dimethylcyclopenta-1,3-diene	3.58	−51.3
5-propyl -1,4-dimethylcyclopenta-1,3-diene	3.46	−56.5
5-butyl-1,4-dimethylcyclopenta-1,3-diene	3.54	−62.1

**Table 6:** Adsorption distances and energies for pyrrole compounds adsorbed on the pristine graphene surface.

Compound	Distance, *d* (Å)	E*_ads_* (kJ mol^−1^)
1,2,5-trimethylpyrrole	3.47	−55.3
1-ethyl-2,5-dimethylpyrrole	3.33	−54.9
1-propyl-2,5-dimethylpyrrole	3.33	−61.0
1-butyl-2,5-dimethylpyrrole	3.31	−66.3

**Table 7:** Information on the best linear fit with intercept equal to zero passing through the data related to the adsorption energy as a function of the number of atoms in the single molecules studied reported in Figure 6.

Compound	Slope	Standard Error	*R* ^2^
Alkanes	−2.3522	0.0426	0.9990
Pyrrolidines	−1.9616	0.0142	0.9998
Pyrroles	−2.5122	0.1050	0.9948
Cyclopentanes	−1.9371	0.0194	0.9997
Cyclopentadienes	−2.4005	0.0624	0.9980

## Data Availability

Data are contained within the article.
